# *Menisporopsisaquatica* sp. nov. (Sordariomycetes, Chaetosphaeriales, Chaetosphaeriaceae), from freshwater habitat in China

**DOI:** 10.3897/BDJ.10.e91008

**Published:** 2022-10-21

**Authors:** Jia-Hao Chen, Dian-Ming Hu, Hai-Yan Song, Zhi-Jun Zhai, Lin Lai, Kang-Hui Lin

**Affiliations:** 1 Bioengineering and Technological Research Centre for Edible and Medicinal Fungi, Jiangxi Agricultural University, Nanchang, China Bioengineering and Technological Research Centre for Edible and Medicinal Fungi, Jiangxi Agricultural University Nanchang China; 2 Jiangxi Key Laboratory for Conservation and Utilization of Fungal Resources, Jiangxi Agricultural University, Nanchang, China Jiangxi Key Laboratory for Conservation and Utilization of Fungal Resources, Jiangxi Agricultural University Nanchang China; 3 College of Bioscience and Bioengineering, Jiangxi Agricultural University, Nanchang, China College of Bioscience and Bioengineering, Jiangxi Agricultural University Nanchang China; 4 Jiangxi Forest Fungi Resources Comprehensive Development Engineering Research Center, Jiangxi Environmental Engineering Vocational College, Ganzhou, China Jiangxi Forest Fungi Resources Comprehensive Development Engineering Research Center, Jiangxi Environmental Engineering Vocational College Ganzhou China; 5 Key Laboratory of Crop Physiology, Ecology and Genetic Breeding (Jiangxi Agricultural University), Ministry of Education of the P.R. China, Nanchang, China Key Laboratory of Crop Physiology, Ecology and Genetic Breeding (Jiangxi Agricultural University), Ministry of Education of the P.R. China Nanchang China

**Keywords:** agamotype, Ascomycota, freshwater fungi, menisporopsis, taxonomy

## Abstract

**Background:**

Freshwater fungi are an integral part of freshwater ecosystems. They promote the carbon cycle of the ecosystem by decomposing wood substrates. *Menisporopsis* is a fungal genus of Chaetosphaeriales in Sordariomycetes, which has been commonly collected from aquatic and marine environments. Most species of this genus are saprophytes.

**New information:**

Here, a new freshwater hyphomycetous fungus, *Menisporopsisaquatica*, reported from submerged rotten wood samples collected in a stream in Zhejiang Province, south-eastern China. The new species is characterised by hyaline conidia appendiculate with 1-2 setulae at each end and synnematous conidiophores growing closely around a black central seta. Molecular phylogeny of *Menisporopsis* was studied using a combined two-loci dataset, including the internal transcribed spacer sequences (ITS) and the nuclear ribosomal large subunit gene sequences (nrLSU). The new species is illustrated and a synopsis of the *Menisporopsis* species is presented in this paper.

## Introduction

The genus *Menisporopsis* S. Hughes (Ascomycota, Sordariomycetes, Chaetosphaeriales, Chaetosphaeriaceae) was first introduced by Hughes (1952) with *Menisporopsistheobromae* S. Hughes as the type species, which was isolated from decaying leaves of *Theobromacacao* L. (Malvaceae) in Ghana. The members of *Menisporopsis* are readily recognised by fungi with pigmented, synnematous conidiophores growing around a central, simple, dark brown seta. The conidiogenous cells are phialidic, producing lunate to falcate, 0- to 1-septate conidia with one to several setulae inserted at the ends or irregularly. The second species, *M.novae-zelandiae* S. Hughes & W.B. Kendr, was described by Hughes and Kendrick (1968). Later, eleven species were included in *Menisporopsis*, i.e. *M.profusa* (Pirozynski and Hodges 1973), *M. pirozynskii* (Varghese and Rao 1978), *M.ludoviciana* (Kirk and Sutton 1985), *M.pleiosetosa* (Rao and Hoog 1986), *M.multisetulata* (Tsui et al. 1999), *M.trisetulosa* (Siboe et al. 1999), *M.anisospora* (Castañeda-Ruiz et al. 2001), *M.kobensis* (Matsushima 2003), *M.pandanicola* (Tibpromma et al. 2018), *M.dushanensis* (Lin 2019) and *M.breviseta* (Lin 2019). A total of thirteen species of *Menisporopsis* have been reported so far. *Menisporopsisludoviciana*, combined *Chaetopsinaludoviciana* J.L. Crane & Schokn, exhibits a branched seta and the conidiophores do not surround the bristles; thus it is distinct from the other twelve species of the genus (Kirk and Sutton 1985). Based on morphology, *M.ludoviciana* (Kirk and Sutton 1985) should belong to the genus *Vermiculariopsiella* Bender (Castañeda-Ruiz et al. 1997). Tsui et al. (1999) and Castañeda-Ruiz et al. (2001), therefore, excluded *M.ludoviciana* from *Menisporopsis*.

Based on the phylogenetic tree and morphology preliminary studies, Menisporopsis, Codinaea, Codinaeopsis, Menispora and Thozetella formed a robust clade within Chaetosphaeriaceae (Réblová and Seifert 2008). During a long-term investigation of freshwater fungi in China (Hu et al. 2012, Huang et al. 2016, Song et al. 2018, Song et al. 2020, Li et al. 2021), a fungus was collected and described as a new species in this paper.

## Materials and methods

### Morphological examination, Isolation and cultivation

Specimens of submerged decaying wood were collected from a stream in Qianjia Lou Brook, Daoxu Town, Shangyu District, Shaoxing City, Zhejiang Province (30.049834N, 120.779092E, altitude 11 m). Samples were taken to the laboratory in sample bags and incubated in plastic boxes at 25°C for two weeks, allowing the formation of fungifruiting bodies on decaying wood to grow under high humidity. Fungal fruiting bodies were examined with a Nikon Ni dissecting microscope. Observations and photographs were made from materials mounted in water with a Nikon Ni compound microscope (Hu et al. 2011). Pure cultures were obtained using a single spore isolation following the method described by Liu et al. (2010). The fungal conidial isolates were cultured on potato dextrose agar (PDA) in a 25℃ incubator to obtain the pure strain. The dried specimens were deposited in the Herbarium of Fungi, Jiangxi Agricultural University, Nanchang, China (HFJAU).

### DNA extraction, sequencing and phylogenetic analyses

Genomic DNA was extracted from pure fungal mycelium growning on PDA following the method described by Hu et al. (2011). We amplified two nrDNA regions: the internal transcribed spacer region of ribosomal DNA (ITS) with the primer pair ITS1 & ITS4 (White et al. 1989) and the nuclear ribosomal large subunit DNA (nrLSU) with primer pair LROR & LR5 (Vilgalys and Hester 1990). The PCR products were sequenced by the same primers used for PCR at Changsha Branch of Tsingke Biotechnology Co., Ltd.

We generated four novel sequences (OM049834, OM049835, OM049836, OM049837) and retrieved twenty-six sequences from GenBank (Table 1). Alignments for each locus were done with MAFFT v.7.307 online version (Katoh and Standley 2016). The Maximum Likelihood (ML) phylogenetic analyses were produced with RAxML v.7.2.6 (Stamatakis and Alachiotis 2010) using a GTRGAMMA substitution model with 1000 bootstrap replicates and evaluated by bootstrap support (MLBS).

For Bayesian Inference analysis, the best-fit model of evolution was determined using MrModelTest v.2 (Nylander 2004). Posterior probabilities (PP) (Zhaxybayeva and Gogarten 2002) were determined by Markov Chain Monte Carlo sampling (BMCMC) in MrBayes 3.0b4 (Huelsenbeck and Ronquist 2001). Two parallel runs with six simultaneous Markov chains were run for 1,000,000 generations, with trees sampled every 100 generations. The first 25% were deleted as burn-in and the posterior probabilities were calculated, based on the remaining trees. The novel taxonomic descriptions and nomenclature were deposited in MycoBank (http://www.mycobank.org/).

### Phylogenetic results

Four new sequences from the strain of a new taxon (OM049834, OM049835, OM049836, OM049837) included two nrLSU and two ITS sequences. The phylogenetic tree of the *Menisporopsis* was constructed, based on the two-loci analysis (Fig. 2). The genetic relationships of a new taxon and other related species were shown. The concatenated aligned dataset comprised twelve species isolates from six *Menisporopsis* species and the isolate of *Leptosporellaarengae* and *Leptosporellabambusae* (Sordariomycetes) as the outgroups. The dataset including alignment gaps comprised 1408 characters: 568 for ITS and 840 for nrLSU. The combined dataset ML tree with bootstrap support values (MLBS) and Bayesian posterior probabilities (BPP) indicates some well-supported clades, with the *Menisporopsisaquatica* strain forming the well-supported clade (MLBS = 90%, BPP = 0.75) with other *Menisporopsis* species.

## Taxon treatments

### Menisporopsis aquatica sp. nov.

4CD2C4CE-0DAA-52B7-A64D-65530EC5EE69

842332

#### Materials

- **Type status:** Holotype. **Occurrence:** recordedBy: Jia-Hao Chen; individualCount: 2; occurrenceID: D16AC014-DFE2-504A-BF1B-607B53CA9FA7; **Taxon:** scientificName: *Menisporopsisaquatica*; acceptedNameUsage: *Menisporopsisaquatica* J.H. Chen, H.Y. Song & D.M. Hu, 2022, sp. nov; parentNameUsage: *Menisporopsistheobromae* S. Hughes 1952; kingdom: Fungi; phylum: Ascomycota; class: Sordariomycetes; order: Chaetosphaeriales; family: Chaetosphaeriaceae; genus: Menisporopsis; specificEpithet: aquatica; taxonRank: species; verbatimTaxonRank: species; scientificNameAuthorship: J.H. Chen, H.Y. Song & D.M. Hu; **Location:** continent: Asia; country: China; stateProvince: Zhejiang; county: Shaoxing; municipality: Shangyu; locality: Qianjia Lou Brook; verbatimElevation: 11 m; locationRemarks: Label transliteration: " Zhejiang, Qianjia Lou Brook, 27/10/2020, Chen Jia-Hao"; [浙江省绍兴市上虞区钱家溇小溪，27/10/2020，陈家豪]; verbatimCoordinates: 30.0498 N, 120.7790 E; decimalLatitude: 30.0498; decimalLongitude: 120.7790; georeferenceProtocol: label; **Identification:** identifiedBy: Jia-Hao Chen and Dian-Ming Hu; dateIdentified: 2020; **Event:** samplingProtocol: collecting; eventDate: 27/10/2020; year: 2020; month: 10; day: 27; habitat: Freshwater; **Record Level:** type: PhysicalObject; language: en; rightsHolder: Dian-Ming Hu; institutionID: HFJAU 10038; collectionID: SXC27; institutionCode: the Herbarium of Fungi, Jiangxi Agricultural University (HFJAU); collectionCode: Fungi; ownerInstitutionCode: the Herbarium of Fungi, Jiangxi Agricultural University (HFJAU); basisOfRecord: PreservedSpecimen

#### Description

**Figure 1.**
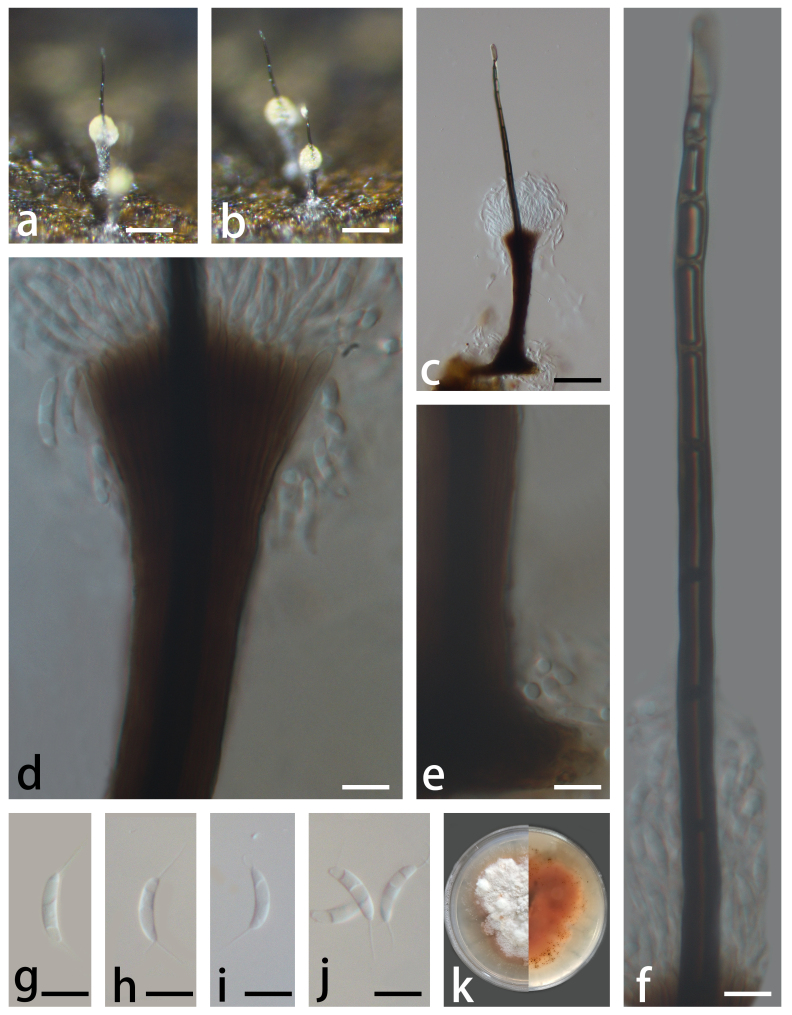
*Menisporopsisaquatica* (HFJAU 10038, Holotype) **a-b** conidiophores and conidia on submerged wood; **c** conidiophores with seta; **d** apex of the conidiophore with developing conidia; **e** base of the conidiophore; **f** seta; **g-j** conidia; **k** colony on PDA. Scale bars: a-b = 100 µm, c = 50 µm, d-j = 10 µm.

Colonies on submerged rotten wood effuse, scattered, pale yellow to pale brown. Mycelium partly immersed, consisting of branched, septate, smooth, thin- to thick-walled, composed of brown hyphae. **Asexual morph**: Stroma and hyphopodia absent. Setae simple, central, solitary, erect, straight, subulate, unbranched, dark brown, 10–12 septate, smooth, thin to thick-walled, swollen at the apex, the lower part of setae encased by numerous tightly compacted conidiophores, 170–324 μm long, 4.8–7.6 μm wide at the base. Conidiophores macronematous, synnematous, brown, smooth, thin-walled, separate, unbranched, cylindrical, erect, straight or slightly flexuous, up to 106 μm long, upper part 1.7–3.5 μm thick. Conidiogenous cells monophialidic, integrated, terminal, pale brown, cylindrical, with collarettes phialides. Conidia acrogenous, semi-endogenous, appendiculate with 6–12 μm long setulae at each end, aggregated into slimy masses at the apex of the synnemata, aseptate, curved, lunate or fusiform, hyaline, smooth, 14–20 μm (av. = 16.5 μm, n = 30) long, 2.4–3.3 μm (av. = 2.7 μm, n = 30) wide. **Sexual morph**: Undetermined (Fig. 1).

##### Etymology

Refers to the fungal freshwater habitat.

##### Notes

*Menisporopsisaquatica* is characterised by synnematous conidiophores growing around a black central seta, which fits well with the genus concept of *Menisporopsis* (Hughes 1952). The phylogenetic results showed that our collection clustered with other *Menisporopsis* species and formed a robust clade.

**Figure 2.**
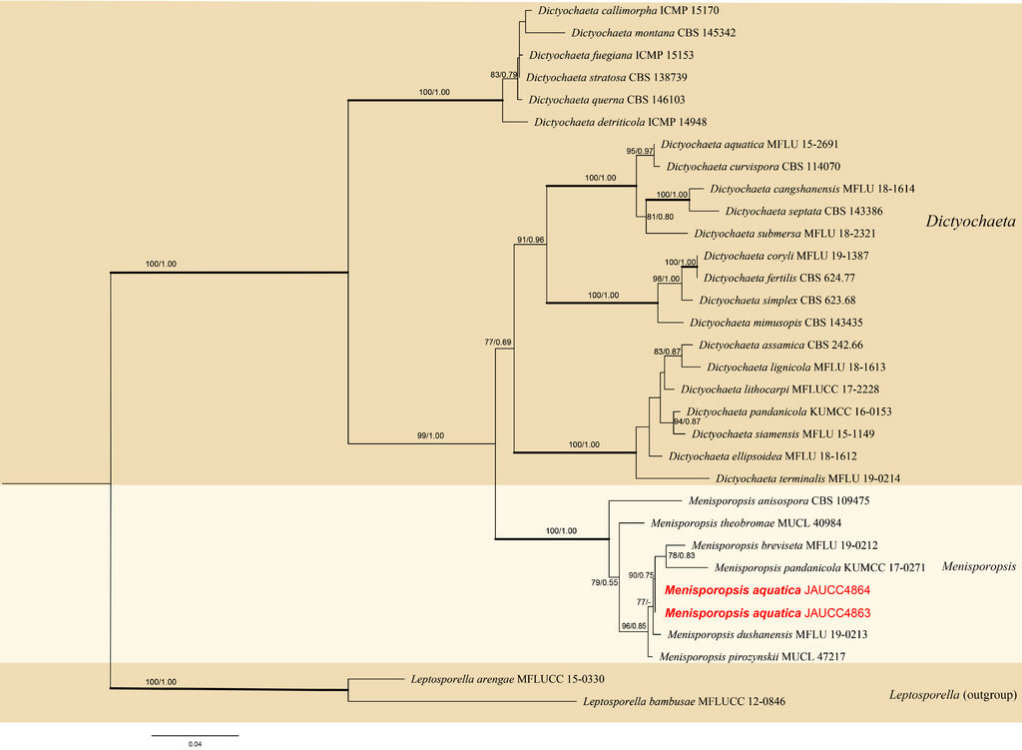
Phylogenetic tree (RAxML) obtained from the DNA sequence data of ITS and LSU sequences of 32 strains showing taxa in *Menisporopsis* and *Dictyochaeta*. The new isolates are shown in bold, red. The MP and ML bootstrap values (BS) ≥ 75% and Bayesian posterior probabilities (PP) ≥ 0.95 are presented at the nodes. The scale bar shows the number of estimated mutations per site. The tree was rooted to *Leptosporellaarengae* (MFLUCC 15-0330) and *Leptosporellabambusae* (MFLUCC 12-0846).

*Menisporopsisaquatica* is close to *Menisporopsisbreviseta* and *Menisporopsispandanicola* in the phylogenetic tree (Fig. 2). *Menisporopsisaquatica* is also morphologically similar to *M.breviseta* in having lunate or fusiform, hyaline conidia appendiculate with 1–2 setulae at each end. However, *M.aquatica* differs from *M.breviseta* by its longer setae (*M.aquatica* 170–324 × 4.8–7.6 μm vs. *M.breviseta* 95–190 × 2.7–5.4 μm) and the longer setulae (*M.aquatica* 6-12 μm vs. *M.breviseta* 4-9 μm). *Menisporopsisaquatica* is morphologically similar to *M.pandanicol*, but differs in its shorter setae (*M.aquatica* 170–324 × 4.8–7.6 μm vs *M.multisetulata* 344–375 × 7–10.5 μm). *Menisporopsisaquatica* can be distinguished from other *Menisporopsis* species by its conidia with 1–2 setula at each end (Table 2).

## Supplementary Material

XML Treatment for Menisporopsis aquatica

## Figures and Tables

**Table 1.** Isolates included in the phylogenetic analyses.

Species	Strains	Status^1^	GenBank accession numbers	
			LSU	ITS
Dictyochaetaaquatica	MFLU 15-2691	T	NG_067563	NR_158452
Dictyochaeta assamica	CBS 242.66		MH870426	MH858788
Dictyochaeta callimorpha	ICMP 15170		MT454500	MT454485
Dictyochaeta cangshanensis	MFLU 18-1614		NG_068636	NR_168801
Dictyochaeta coryli	MFLU 19-1387		NG_073859	NR_171096
Dictyochaeta curvispora	CBS 114070		—	MH862954
Dictyochaeta detriticola	ICMP 14948		MT454501	MT454486
Dictyochaeta ellipsoidea	MFLU 18-1612		NG_068633	NR_168798
Dictyochaeta fertilis	CBS 624.77		—	AF178540
Dictyochaeta fuegiana	ICMP 15153		—	MT454487
Dictyochaeta lignicola	MFLU 18-1613		NG_068634	NR_168799
Dictyochaeta lithocarpi	MFLUCC 17-2228		NG_073858	NR_171095
Dictyochaeta mimusopis	CBS 143435		MH107935	MH107888
Dictyochaeta montana	CBS 145342		MT454502	NR_172307
Dictyochaeta pandanicola	KUMCC 16-0153	T	MH376710	MH388338
Dictyochaeta querna	CBS 146103		MT454504	MT454490
Dictyochaeta septata	CBS 143386		MH107936	MH107889
Dictyochaeta siamensis	MFLU 15-1149		NG_059142	NR_154016
Dictyochaeta simplex	CBS 623.68		MH878418	MH859497
Dictyochaeta stratosa	CBS 138739		MT454505	NR_172308
Dictyochaeta submersa	MFLU 18-2321		NG_068635	NR_168800
Dictyochaeta terminalis	MFLU 19-0214	T	NG_067903	NR_166297
Leptosporellaarengae	MFLUCC 15-0330		MG272246	MG272255
Leptosporellabambusae	MFLUCC 12-0846		KU863122	KU940134
Menisporopsis anisospora	CBS 109475	T	MH874421	MH862827
Menisporopsisaquatica	JAUCC4863		OM049834	OM049837
Menisporopsisaquatica	JAUCC4864		OM049835	OM049836
Menisporopsisbreviseta	MFLU 19-0212	T	NG_070469	NR_166296
Menisporopsisdushanensis	MFLU 19-0213	T	NG_070470	NR_166299
Menisporopsispandanicola	KUMCC 17-0271	T	MH376726	MH388353
Menisporopsis pirozynskii	MUCL 47217		MW984561	MW984579
Menisporopsistheobromae	MUCL 40984		MW984563	MW984581

**Table 2.** Synopsis of the *Menisporopsis* species described to date.

Species	Setae	Conidiomata	Conidia		Setulae	
	（μm）	（μm）	Shape	Size (μm)	Number	Size (μm)
*M.anisospora*	200–425 × 10–12	300–550 × 60–80	Allantoid to irregular, truncate at base	17.0–30.0 × 2.0–6.0	1 at each end; 1-3 lateral	Apical: 4.0–11.0; basal: 3.0-10.0
*M.aquatica*	170–324 × 4.8–7.6	106 × 1.7–3.5	Lunate or fusiform	14.9–19.9 × 2.4–3.3	1-2 basal; 1 apical	6–12
*M.breviseta*	95–190 × 2.7–5.4	182 × 2.5–4.6	Cylindrical or fusiform	14.2–24.3 × 2.4–3.6	1-2 setulae at each end	4–9
*M.dushanensis*	207–455 × 5–10.5	147 × 2.5–6	Cylindrical or fusiform	14.0–21 × 3–4	1-2 setulae at each end	3–11
*M.kobensis*	195–275 × 6.0–7.5	None	Allantoid to lunate	16.0–32.0 × 3.0–5.0	1 at each end	6.0–10.0
*M.multisetulata*	300–500 × 6–10	180–220 × 22–40	Allantoid	12.0–19.0 × 2.5–4.0	3–4 basal; 2–3 apical	7.0–10.0
*M.pandanicola*	344–375 × 7–10.5	85–100 × 14.5–23	Cylindrical, lunate	8–29 × 0.5–2	(1–)2 setula at each end	4–12
*M pirozynskii*	132–450 × 3–14	35–250 × 12–60	Cylindrical to lunate	12.0–20.5 × 2.0-4.5	1–3 basal; 2 apical	2.0–12.0
*M.pleiosetosa*	100–300 × 3–4	≤ 250 × 30–40	Ellipsoidal, truncated at base	12.0–18.0 × 4.0–5.0	2–4 basal; 1 apical	≤ 6.0
*M.profusa*	150–425 × 4.5–9.0	60–225 × 12.5–18	Cylindrical, allantoid to lunate	7.0–15.0 × 1.2–2.5	1 at each end	3.0–6.0
*M.theobromae*	105–460 × 4.5–7.5	55–170 × 12–35	Lunate to falcate	11.0–20.0 × 1.5–4.0	1 at each end	5.0–10.0
*M.trisetulosa*	250–460 × 5.5–7.5	None	Allantoid	12.0–20.0 × 2.0	2 basal; 1 apical	ca. 10.0
